# Enhancing the Hydrogen Evolution Reaction Activity of Platinum Electrodes in Alkaline Media Using Nickel–Iron Clusters

**DOI:** 10.1002/anie.202000383

**Published:** 2020-04-30

**Authors:** Song Xue, Richard W. Haid, Regina M. Kluge, Xing Ding, Batyr Garlyyev, Johannes Fichtner, Sebastian Watzele, Shujin Hou, Aliaksandr S. Bandarenka

**Affiliations:** ^1^ Physics of Energy Conversion and Storage, Physik-Department Technische Universität München James-Franck-Strasse 1 85748 Garching Germany; ^2^ Catalysis Research Center TUM Ernst-Otto-Fischer-Strasse 1 85748 Garching Germany

**Keywords:** alkaline water electrolysis, electrocatalysis, hydrogen evolution reaction (HER), nickel–iron clusters, platinum

## Abstract

Herein, we demonstrate an easy way to improve the hydrogen evolution reaction (HER) activity of Pt electrodes in alkaline media by introducing Ni–Fe clusters. As a result, the overpotential needed to achieve a current density of 10 mA cm^−2^ in H_2_‐saturated 0.1 m KOH is reduced for the model single‐crystal electrodes down to about 70 mV. To our knowledge, these modified electrodes outperform any other reported electrocatalysts tested under similar conditions. Moreover, the influence of 1) Ni to Fe ratio, 2) cluster coverage, and 3) the nature of the alkali‐metal cations present in the electrolyte on the HER activity has been investigated. The observed catalytic performance likely originates from both the improved water dissociation at the Ni–Fe clusters and the subsequent optimal hydrogen adsorption and recombination at Pt atoms present at the Ni–Fe/Pt boundary.

## Graphical abstract

**HER we go**: An improved alkaline HER performance was achieved by modifying Pt(111) and nanostructured Pt electrocatalysts with nickel‐iron clusters. The activity was optimized by varying the Ni:Fe ratio, the coverage of the metal cluster, and the electrolyte composition. This improvement yields a more favorable balance between benefiting water dissociation and preventing *OH “poisoning”.

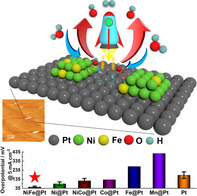

Performing water splitting in alkaline media has many advantages over its acidic analogue, such as increased stability of the electrocatalysts and higher cost efficiency.^1^, ^2^, ^3^, ^4^ However, improving the sluggish hydrogen evolution reaction (HER) kinetics at high pH values is still a very critical step towards the profitable realization of alkaline electrolyzers. Various hypotheses have been developed to explain the slow HER kinetics in alkaline media. The Trasatti^5^ and Norskov^6^ groups proposed a volcano‐type relationship between experimental HER current densities and theoretical hydrogen adsorption energies mainly for acidic media. Based on the observation of positive peak shifts in the hydrogen underpotential deposition region of Pt(100) and Pt(110) electrodes, Sheng et al.^7^ suggested that the hydrogen binding energies strengthened by hydroxide anions are responsible for the decreased reaction rate in an alkaline environment. These shifts could, however, also be ascribed to weakened OH‐adsorption energies as a result of the surrounding alkali metal cations.^8^, ^9^, ^10^ Evidently, the sluggish reaction kinetics at higher pH values cannot solely be explained by hydrogen adsorption energies. Improving the kinetics of the rate‐determining step, water dissociation, which is correlated with the OH‐adsorption energy, is nowadays recognized as a promising strategy to improve the HER activity in alkaline solutions.^4^, ^11^ Moreover, Koper et al.^12^ recently reported that the electric field formed at the electrochemical interface is “strengthened” in the alkaline environment. As a result, the charge transfer barrier across the electrical double layer increases and causes limited HER kinetics. Although still under debate, these observations collectively dominate the state‐of‐the‐art design approaches for the alkaline HER catalysts.

Guided by these considerations, Pt surfaces modified with non‐noble metal hydroxide clusters have been examined: the Pt surfaces offer optimal hydrogen adsorption energy, whereas the non‐noble metal hydroxides support water dissociation.^13^, ^14^ According to the Brønsted‐Evans‐Polanyi principle, the ability of a catalyst to dissociate water is correlated with the OH‐adsorption energy.^13^ However, too strong OH‐adsorption is undoubtedly ineligible, as active sites can get blocked.^14^ To‐date, Pt surfaces modified with Ni‐hydroxide clusters are recognized as the most active HER electrocatalyst. The current understanding of the origin of such high activity is that the Ni hydroxides provide an optimal water dissociation, while at the same time Pt promotes H_2_ generation.^14^ Moreover, various studies on Ni–Fe hydroxides, which are among the best catalysts for the oxygen evolution reaction (OER), showed that relative to pure Ni hydroxide films, the addition of Fe can enhance their conductivity more than 30 times.^15^ Furthermore, X‐ray absorption spectroscopy and coulometric titration indicated that the addition of Fe can increase the oxidation state of Ni atoms in its vicinity,^16^, ^17^ which implies an improved water dissociation ability due to the increased *OH binding energy (* corresponds to an adsorption site).^18^ Herein, we introduced both Fe and Ni clusters to a Pt surface and investigated the resulting HER activity in various alkaline electrolytes. The highest activity was observed in aqueous KOH, the overpotential needed to achieve a current density of 10 mA cm^−2^ was approximately 70 mV, which corresponds to the highest reported activity for the HER in alkaline media under similar conditions.

We start our analysis with the observation that introducing Ni and Fe to the Pt(111) surface (NiFe@Pt(111)) improves the HER performance with respect to an unmodified Pt(111) electrocatalyst and shifts the potential of the OH‐adsorption peak towards a more negative value, as visible in the polarization curves and cyclic voltammograms (CVs) depicted in Figure 1. Consistent with previous studies, Ni‐modified Pt(111) (Ni@Pt(111)) electrodes show an improved performance compared to plain Pt(111). Thereby, the overpotential at 10 mA cm^−2^ was approximately 128 mV, comparable to 130 mV reported by Feliu et al.^19^ and 124 mV reported by Yu et al.^20^ The overpotential for the NiFe@Pt(111) catalyst, however, is significantly decreased to around 70 mV, approaching that of Pt(111) in 0.1 m HClO_4_ (Figure 1 A). The corresponding CVs are depicted in Figure 1 B. The CVs of plain Pt(111) in acidic and alkaline media can be divided into three regions: the adsorption/desorption of hydrogen (H_ads/des_), the double layer, and the adsorption/desorption of hydroxide species (OH_ads/des_). After modification with the Ni clusters, an additional pair of peaks appears at around 0.70 V_RHE_ and 0.53 V_RHE_ (V_RHE_ versus reversible hydrogen electrode). These peaks can be assigned to the adsorption and desorption of OH species, likely occurring at the Ni sites, but a possible contribution from *O formation should not be excluded.^19^ The CVs are identical to those in the literature^12^, ^13^, ^19^ and confirm the presence of the Pt(111) surface during the modification procedure. Typical CVs of NiFe@Pt(111) electrodes resemble that of Ni@Pt(111); however, the OH adsorption peaks are slightly negatively shifted (see the direct comparison in Figure S1 in the Supporting Information). This shift implies that the adsorption of OH is promoted in the case of the NiFe@Pt(111) electrodes. In this regard, the binding energy of the OH species increases^21^ as the cluster coverage on the Pt surface becomes comparable for the Ni@Pt(111) and NiFe@Pt(111) samples (Figure S2). This observation is supported by recent findings from Zhao et al.,^18^ and suggest that the added Fe pulls partial electrons from Ni sites and increases its electron affinity, which may facilitate the OH adsorption.

**Figure 1:**
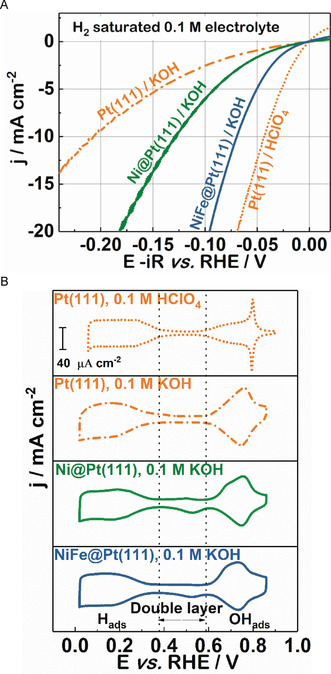
A) Typical polarization curves of Pt(111), Ni@Pt(111) and NiFe@Pt(111), recorded in H_2_‐saturated 0.1 m aqueous KOH at a scan rate of 50 mV s^−1^ and a rotational speed of 1600 rpm. A polarization curve of a Pt(111) electrode recorded in H_2_‐saturated HClO_4_ is shown for comparison. All polarization curves are 85 % iR corrected. B) Corresponding CVs of Pt(111), Ni@Pt(111), and NiFe@Pt(111), recorded in Ar‐saturated electrolyte at a scan rate of 50 mV s^−1^. Orange: Pt(111); green: Ni@Pt(111); navy blue: NiFe@Pt(111).

The extent of the observed activity enhancement appears to depend on the Ni to Fe ratio of the clusters (Figure S3 A). Several ratios were tested to optimize the performance. In general, all investigated ratios showed an improved HER performance compared to Ni@Pt(111). The highest activity enhancement was observed at a Ni:Fe ratio of 3:1, as determined by X‐ray photoelectron spectroscopy (XPS) measurements (Figure S3 B).

The HER performance of the NiFe@Pt(111) appears to be also closely associated with the Ni–Fe cluster coverage on Pt(111). To investigate this effect, three different coverages were achieved by adjusting the concentration of the precursor solution and investigated using scanning tunneling microscopy (STM), as shown in Figure 2 A–C. At a relatively low concentration, clusters of up to 3 nm in height can be identified either individually or as agglomerates on the Pt surface (see NiFe*@Pt(111) in Figure 2 A and Figure S4 B). To confirm the presence of the Ni–Fe clusters, Au‐coated glass plates were coated using an identical precursor concentration and imaged via scanning electron microscopy (SEM). Similar structures can be observed (Figure S5), although the SEM sensitivity to height is limited. In addition to these microscopy images, XPS confirms the presence of Ni and Fe (Figure S3 B,C). Thus, we assign the clusters to be Ni–Fe rather than Pt, since pure Pt(111) possesses a rather smooth flat surface (Figure S4 A) and non‐noble metals (hydroxides) typically follow a Volmer–Weber growth that leads to the formation of 3D clusters.^13^, ^19^ With increasing precursor solution concentration, shown as NiFe@Pt(111) in Figure 2 B and Figure S4 C, the clusters follow a 3D growth mechanism, leading to cluster agglomerates of about 6 nm in height. Full surface coverage can be observed with a further increase in concentration, leading to a smooth Ni–Fe film (see NiFe**@Pt(111) in Figure 2 C and Figure S4 D). The corresponding HER performances are compared in Figure 2 D and S6. The activity order observed is as follows: NiFe@Pt(111) > NiFe*@Pt(111) > NiFe**@Pt(111) > pure Pt(111). The drop in the kinetic activity of the highest Ni–Fe coverage could be caused by the obstruction of active sites on the Pt surface.^22^

**Figure 2:**
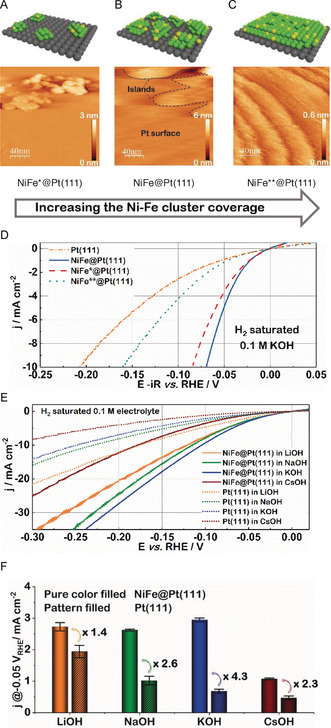
A)–C) Schematic representations and STM images of the NiFe@Pt(111) electrode surface at different Ni–Fe cluster coverages (constant Ni:Fe ratio of 3:1). D) Corresponding HER curves, recorded in H_2_‐saturated 0.1 m KOH at a scan rate of 50 mV s^−1^ and a rotational speed of 1600 rpm. All curves are corrected for the ohmic drop (85 %). The activity of plain Pt(111) is shown for comparison. NiFe@Pt(111), NiFe*@Pt(111), and NiFe**@Pt(111) are samples prepared from precursor solutions with standard, 100 times lower, and 1000 times higher concentration, respectively (see details in the experimental section). E) and F) HER activities of Pt(111) and NiFe@Pt(111) electrodes measured in 0.1 m H_2_‐saturated LiOH, NaOH, KOH, and CsOH, respectively. The polarization curves of Pt(111) and NiFe@Pt(111) electrocatalysts (Ni:Fe ratio of 1:1) were recorded in different alkaline solutions recorded at a rotation rate of 1600 rpm and a scan rate of 50 mV s^−1^ (E). The extracted current densities and enhancement factors at a potential of −0.05 V_RHE_ are shown in (F). Error bars represent the standard deviations of at least three independent experiments.

As previously reported by our group, the HER activity of different Pt‐based surfaces can be tuned by changing the type of alkali metal cation present in the electrolyte.^23^, ^24^, ^25^ To investigate this cation effect on the HER activity of NiFe@Pt(111), the performance of the electrodes in LiOH, NaOH, KOH, and CsOH was investigated, as summarized in Figure 2 E. Indeed, the activity enhancement depends on the electrolyte composition.^26^ The HER activity of NiFe@Pt(111) can be increased by a factor of about 3, for example, when using KOH instead of CsOH. When compared to pure Pt(111), the activity of the NiFe@Pt(111) increases by a factor of 4.3, 2.6, and 1.4, in KOH, NaOH, and LiOH, respectively (Figure 2 F). For the CsOH electrolytes, the activity improves by a factor of 2.3; however, the activity is considerably lower than in the other electrolytes. The reason for the different enhancement factors in different electrolytes may be explained by different H‐ and OH‐adsorption energies in the presence of different alkali metal cations. However, a more profound confirmation requires further investigations.

To demonstrate the applicability of Ni–Fe modified Pt surfaces for industrial purposes, Pt/C (TKK) nanoparticles with an average Pt size of approximately 3 nm was modified and tested using the procedures discussed in the experimental section. Indeed, after “Ni–Fe modification”, the commercial catalyst exhibited improved HER activity, as shown in Figure 3 A. The bar chart in Figure 3 B indicates that after the modification with Ni clusters, the overpotential which is necessary to reach the current density of 10 mA cm^−2^ decreases by around 8 %, compared to unmodified commercial Pt/C catalysts. The overpotential is further reduced by about 20 % after introduction of the Ni–Fe clusters. These results confirm that indeed a performance enhancement through modification with Ni–Fe clusters can also be achieved in case of commercial nanostructured materials.

**Figure 3:**
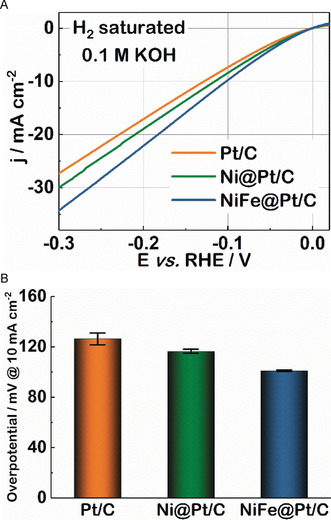
HER performance of commercial Pt/C (TKK) electrocatalyst, before and after modification with Ni and Ni–Fe clusters (Ni@Pt/C and NiFe@Pt/C). A) Polarization curves of pure Pt/C, Ni@Pt/C, and NiFe@Pt/C electrodes, recorded in H_2_‐saturated KOH at a rotational speed of 1600 rpm and a scan rate of 10 mV s^−1^. Loading mass: 20 μg_Pt_ cm^−2^. B) HER overpotentials of commercial Pt/C, Ni@Pt/C, and NiFe@Pt/C electrocatalysts at a current density of 10 mA cm^−2^, derived from the polarization curves shown in (A). Standard deviations were obtained from at least three independent experiments. All current densities for the commercial Pt/C are normalized to the geometrical surface area of the glassy carbon electrode (0.196 cm^2^).

Evidently the enhanced activity originates from an interplay between the Pt catalyst and the deposited Ni–Fe clusters, rather than the Ni–Fe cluster itself or a simple physical superposition of their HER activities, since a significantly worse HER performance was observed in the absence of Pt (Figure 4 A). We assume that, based on the previously mentioned alkaline HER mechanisms,^14^ Ni–Fe clusters are beneficial for the initial water dissociation and subsequently, Pt offers optimal conditions for the hydrogen adsorbate recombination and the hydrogen gas evolution (Scheme 1).

**Figure 4:**
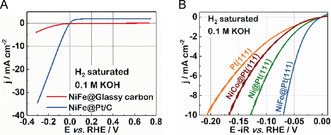
A) Polarization curves of NiFe@Pt/C and pure Ni–Fe, deposited on glassy carbon and recorded in 0.1 m H_2_‐saturated KOH. Scan rate: 10 mV s^−1^. Rotational speed: 1600 rpm. B) Typical polarization curve of NiCo@Pt(111) recorded in 0.1 m H_2_‐saturated KOH. Scan rate: 50 mV s^−1^. Rotational speed: 1600 rpm. The polarization curves of Pt(111), Ni@Pt(111), and NiFe@Pt(111) are shown for comparison. All curves are corrected for the IR‐drop (85 %).

**Scheme 1:**
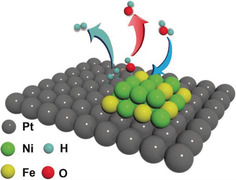
Proposed mechanism of HER on NiFe@Pt(111) electrodes in alkaline media. Ni–Fe cluster improves the initial water dissociation step (as indicated by the blue arrow), and Pt promotes the following hydrogen adsorbate recombination and hydrogen gas evolution (as shown by the jade arrow).

A further aspect could be a cooperative mechanism, where adding Fe assists Ni clusters to dissociate water. This assumption is supported by the increased *OH binding energies of Ni–Fe clusters, since an earlier onset of the OH_ads_ region (Figure S7) as well as a correspondingly larger surface coverage area (Figure S8) was observed for NiFe@Pt(111) compared to Ni@Pt(111). To further verify this hypothesis, the HER activity of Pt(111) modified with Ni–Co clusters (NiCo@Pt(111)) was measured under the same experimental conditions as NiFe@Pt(111). Hereby, a further increase in *OH binding energy is obtained on NiCo@Pt(111) (Figure S7 and S8). However, as depicted in Figure 4 B, NiCo@Pt(111) displays an even worse HER performance than Ni@Pt(111). Thus, although the increased adsorption of OH species induced by the addition of Co can help to dissociate the water, too strong OH interactions “poison” the Ni–Co clusters for the subsequent reaction steps. Hence, the NiFe@Pt provides a more favorable balance between benefiting the water dissociation and preventing *OH “poisoning”. Moreover, these experiments confirm that the *OH binding energy may be one of the important descriptors for alkaline HER (Figure S9). Thereby, a too weak *OH binding energy causes a slow water dissociation, whereas a too strong *OH binding energy leads to *OH “poisoning”. Adjusting the electronic band structure of Ni to find a more favorable balance appears to be a feasible way to further improve the alkaline HER performance.

In summary, the HER activity of Pt in alkaline media can be significantly enhanced by modifying its surface with the Ni–Fe clusters. The degree of this improvement depends on the ratio of Ni to Fe, the surface coverage, and the electrolyte composition. We assume that this improved activity is due to a more favorable balance between benefiting water dissociation and preventing *OH “poisoning”. With this enhancement, we further boost the alkaline HER efficiency, shifting it noticeably closer to the activities in acidic solution.

## Conflict of interest

The authors declare no conflict of interest.

## Supporting information

As a service to our authors and readers, this journal provides supporting information supplied by the authors. Such materials are peer reviewed and may be re‐organized for online delivery, but are not copy‐edited or typeset. Technical support issues arising from supporting information (other than missing files) should be addressed to the authors.

Supplementary
