# Supplementary material for: Enhancing the Hydrogen Evolution Reaction Activity of Platinum Electrodes in Alkaline Media Using Nickel–Iron Clusters
Source: Angew Chem Int Ed Engl. 2020 Apr 30;59(27):10934–8. doi: 10.1002/anie.202000383 (PMC7318285; doi:10.1002/anie.202000383)
Supplement: Supplementary file 1 — Supplementary [file ANIE-59-10934-s001.pdf]

Angewandte Chemie
Eine Zeitschrift der Gesellschaft Deutscher Chemiker

Supporting Information

Enhancing the Hydrogen Evolution Reaction Activity of Platinum Electrodes in Alkaline Media Using Nickel–Iron Clusters

*Song Xue, Richard W. Haid, Regina M. Kluge, Xing Ding, Batyr Garlyyev, Johannes Fichtner, Sebastian Watzele, Shujin Hou, and Aliaksandr S. Bandarenka**

anie_202000383_sm_miscellaneous_information.pdf

Table of Contents

1. Experimental Section........S3

2. Supplementary Figures........S4

Figure S1. CVs of Pt(111), Ni@Pt(111), NiFe@Pt(111) electrodes........S4

Figure S2. HOR and ORR on Ni@Pt/C, NiFe@Pt/C electrodes........S5

Figure S3. RDE voltammograms and XPS results of NiFe@Pt(111) with different ratios of Ni to Fe........S6

Figure S4. STM images of NiFe@Pt(111) with different cluster coverages........S7

Figure S5. SEM images of NiFe@Au-coated glass plates with different cluster coverages........S8

Figure S6. Tafel plots for HER on Pt(111) and NiFe@Pt(111) with different cluster coverages........S9

Figure S7. Comparison of OH_{ads} peaks for Pt(111) modified with Ni, Ni-Fe, Ni-Co clusters........S10

Figure S8. Comparison of OH_{ads} charge for Pt(111) modified with Ni, Ni-Fe, Ni-Co clusters........S11

Figure S9. “Volcano plot” of Pt modified with different metal hydroxide clusters........S12

Figure S10. Current-time plot obtained for the surface-modification process........S13

Figure S11. Polarization curves of Ni@Pt/C and NiFe@Pt/C including both HER and HOR........S14

Figure S12. Stability of NiFe@Pt(111)........S15

3. Supplementary References........S16

1. Experimental Section

Cell preparation. Prior to the measurements, all glassware was carefully cleaned with a piranha solution, 1:3 ratio mixture of H_2O_2 and H_2SO_4 (both Suprapur, Merck, Germany), followed by boiling it in ultrapure water (Evoqua, Germany) several times. A mercury-mercury sulfate electrode (SI Analytics, Germany) was taken as the reference electrode and connected to the working electrolyte by ionically conducting glass. A Pt wire was employed as the counter electrode. All potentials shown in this work are referred to the reversible hydrogen electrode (RHE) scale.

Electrode preparation. To ensure the accuracy of the measurements on single crystalline Pt(111), both bead-type (99.99%, 0.049 cm^2, Icryst, Germany) and disk-type (99.99%, 0.196 cm^2, Mateck, Germany) crystals were used. Prior to the measurements, all single crystals were flame-annealed in an isobutane flame, followed by cooling in a mixture of 1000 ppm CO (4.7, Air Liquide, Germany) and Ar (5.0, Air Liquide, Germany). The surface quality was characterized by cyclic voltammetry experiments in Ar-saturated 0.1M $HClO_4$ (Suprapur, Merck, Germany). Experiments on commercial Pt/C were performed by drop-casting 10 µL of catalyst ink on a glassy carbon electrode with a geometrical area of 0.196 cm^2 (Pine, USA), followed by drying at 400 rpm. The ink was prepared by dispersing 10 mg of commercial catalyst powder (TEC10V20E, Tanaka Kikinzoku, Japan) in a mixture of ultrapure water (3600 µL), isopropanol (1446 µL) and Nafion (30 µL, 5% dispersion in lower aliphatic alcohols and water, Sigma Aldrich, Germany). The ink was placed in an ultrasonic bath for 10 minutes prior to utilization, in order to achieve homogeneous dispersion of the catalyst.

Deposition of Ni and Ni-Fe metal clusters. For the deposition of Ni clusters, 10 µM $NiSO_4$ solution was prepared by dissolving $NiSO_4 \cdot 6H_2O$ (99% Sigma Aldrich) in ultrapure water. For the deposition of Ni-Fe clusters, five different Ni:Fe ratios with an overall concentration of 10 µM, namely 1:1, 3:1, 6:1, 9:1, and 15:1, were prepared by dissolving the respective amounts of $NiSO_4 \cdot 6H_2O$ and $FeSO_4 \cdot 6H_2O$ (99% Sigma Aldrich) in ultrapure water (e.g. 9 µM $NiSO_4$ and 1 µM $FeSO_4$ for 9:1 Ni:Fe ratio).Preparation of the Ni-Co deposition solution followed the same recipe, using $CoSO_4 \cdot 7H_2O$ (99% Sigma Aldrich) as a precursor salt. In order to investigate the effect of different Ni-Fe cluster coverages on the activity, three different precursor concentrations were employed. The precursor solution consisting of 9 µM $NiSO_4$ and 1 µM $FeSO_4$ was taken as a "standard" concentration, and the obtained samples were designated as NiFe. Additionally, 1000 times higher concentration and 100 times diluted precursor solutions were prepared for comparison, and the corresponding samples were designated as NiFe** and NiFe*, respectively.

Deposition of the clusters was performed by dropping either 10 µL or 30 µL of deposition solution on the surface of the Pt(111) bead-type single crystal (10 µL), the Pt(111) disk-type single crystal and the Pt/C-coated glassy carbon electrode (both 30 µL). The electrode was then transferred into Ar-saturated electrolyte (normally, 0.1 M KOH), at a pre-set electrode potential of 0.1 V_{RHE} under a hanging meniscus configuration. After ~30 seconds, the electrode was removed from the electrolyte under the same potential. (**Figure S10**)

Electrochemical measurements. CV measurements were performed in both Ar-saturated 0.1 M $HClO_4$ and KOH (99.99%, Trace Metal Basis, Sigma Aldrich) under a hanging meniscus configuration at a scan rate of 50 mV/s. The HER and hydrogen oxidation reaction (HOR) polarization curves were recorded using a pine RDE 710 instrument (USA) at 1600 rpm in H_2-saturated 0.1 M KOH (HOR see **Figure S2A and S11**). Potentials were controlled by a VSP-300 potentiostat (Bio-Logic, France). Oxygen reduction reaction (ORR) polarization curves were recorded in O_2-saturated 0.1 M KOH (**Figure S2B**). In those activity measurements, several cycles (5-10) of cyclic voltammetry were performed to both achieve a relatively steady curve and exclude the effect of impurities building up in the electrolyte. The stability of NiFe@Pt(111) was also tested by CV measurements in H_2-saturated 0.1 M KOH, in which a glassy carbon rod was used as the counter electrode instead of the Pt wire, to avoid possible Pt deposition effects during cycling (**Figure S12**). It should be noted that all current densities are normalized to the geometrical surface area of the electrode.

STM measurements. STM was performed under ambient conditions using a scanning probe microscope (MultiMode, Veeco Instruments Inc.) controlled by a dedicated software control system (NanoScope, Digital Instruments) and a potentiostat (Universal Bipotentiostat, Veeco Instruments Inc.). STM tips were prepared by mechanically ripping a $Pt_{80}Ir_{20}$ wire (Goodfellow GmbH, Diameter: 0.25 mm). Images were recorded at a tip current of ~1 nA and a tip potential of ~100 mV.

XPS measurements. Chemical analysis via XPS was performed on a setup by SPECS, including a SPECS XR50 X-ray source (Anode: Al, 1487 eV), a SPECS PHOIBOS 150 hemispherical analyzer and SPECS spectrometer. XPS Peak software (version 4.1) was used to fit the obtained spectra by utilizing a Shirley background subtraction and using a mixture of 30% Lorentzian and 70% Gaussian functions for the least squares curve fitting procedure, in which binding energies were initially corrected based on the C-C/C-H peak of adventitious carbon at 284.8 eV in the C1s spectrum.

SEM measurements. SEM samples were prepared following the same procedures as that of NiFe@Pt(111), however, using Au-coated glass plates (1.1 cm × 1.1 cm, Arrandee) as substrates instead of Pt(111) . An NVision40 FE-SEM (Carl Zeiss AG) was used for SEM measurements at an acceleration voltage of 3 kV and at a working distance of ~3.5 mm.

2. Supplementary Figures

Figure S1. CVs of Pt(111), Ni@Pt(111), NiFe@Pt(111) electrodes

In the CVs of Pt(111), Ni@Pt(111) and NiFe@Pt(111), consecutive negative shifts of the OH adsorption peak can be observed after modification, which is even visible at low cluster coverage (**Figure S1A**) and becomes even more apparent at higher cluster coverage (**Figure S1B**).

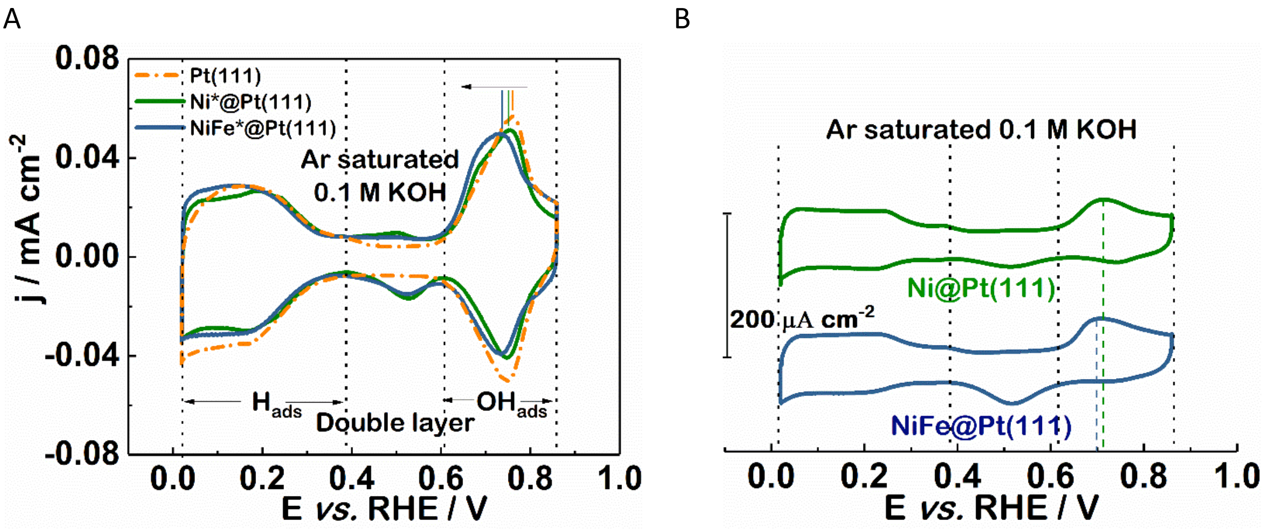

Figure S1. CVs of (A) Pt(111), Ni*@Pt(111) and NiFe*@Pt(111) (i.e. 100 times lower concentration), as well as (B) Ni@Pt(111)/ and NiFe@Pt(111) (i.e. standard concentration), recorded in Ar-saturated 0.1 M KOH. Scan rate: 50 mV s^{-1}.

Figure S2. HOR and ORR on Ni@Pt/C, NiFe@Pt/C electrodes.

It is necessary to determine whether the cluster coverage is significantly influenced by adding Fe, as the importance of Ni cluster coverage on Pt surfaces for HER enhancement has previously been reported by several groups[1,2,3]. For this reason, we firstly compared the results observed from Ni-Fe clusters with those of the optimal Ni cluster coverage reported recently by Feliu et al. [2]. At a potential of -0.05 V_{RHE}, the current density of NiFe@Pt(111) is 5 mA cm^{-2}, almost twice as high as the one obtained with optimal Ni cluster coverage. This indicates that changing the Ni cluster coverage is not sufficient to explain the enhancement. Subsequently, the limiting current densities of HOR and ORR for Ni@Pt/C and NiFe@Pt/C were taken into account, as they could be positively correlated with the exposed active area in HOR[4] and ORR[5] reactions to some degree. The limiting current densities for both Ni@Pt/C and NiFe@Pt/C electrodes were found to decrease similarly. These results imply that the additional introduction of Fe does not affect the cluster coverage of the Pt surface.

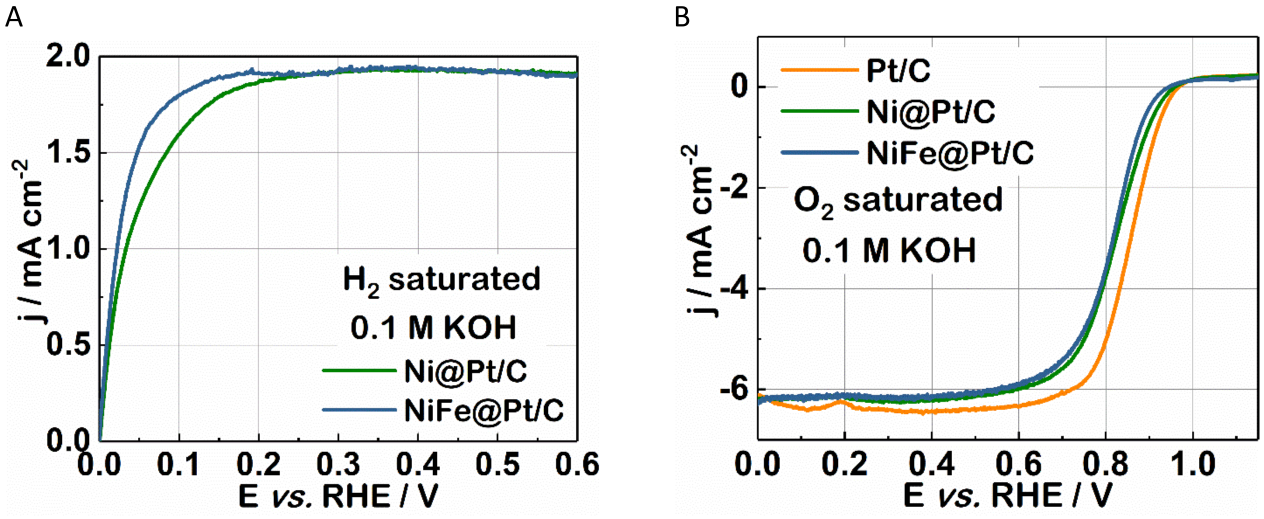

Figure S2. HOR and ORR on Ni@Pt/C, NiFe@Pt/C electrodes. (A) HOR polarization curves of Ni@Pt/C and NiFe@Pt/C electrodes, measured in 0.1 M H_2 saturated KOH electrolyte. Scan rate: 10 mV s^{-1}. Electrode rotated at 1600 rpm. (B) ORR on Pt/C, Ni@Pt/C, NiFe@Pt/C electrodes, measured in 0.1 M O_2 saturated KOH. Scan rate:10 mV s^{-1}. Electrode rotated at 1600 rpm.

Figure S3. RDE voltammograms and XPS results of NiFe@Pt(111) with different ratios of Ni to Fe.

To investigate the effect of the Ni:Fe ratio on HER performance, five different ratios of Ni to Fe were studied. It should be noted that the ratios shown here are not actual ratios, but feeding ratios (**Figure S3A**). In general, all investigated ratios showed an improved HER performance compared to the Ni@Pt(111) electrocatalysts. XPS was used to check the real ratio of Ni to Fe on the Pt surface. According to XPS measurements, deposition from a 9:1 ratio precursor solution results in a 3:1 Ni:Fe ratio of the clusters grown on the Pt(111) surface (**Figure S3B**). A 3:1 ratio precursor solution, in turn, yields a 1:1 Ni:Fe ratio deposition (**Figure S3C**). This discrepancy may stem from the deposition procedure, for instance, due to different ion mobilities of Ni and Fe towards a Pt surface.[6]

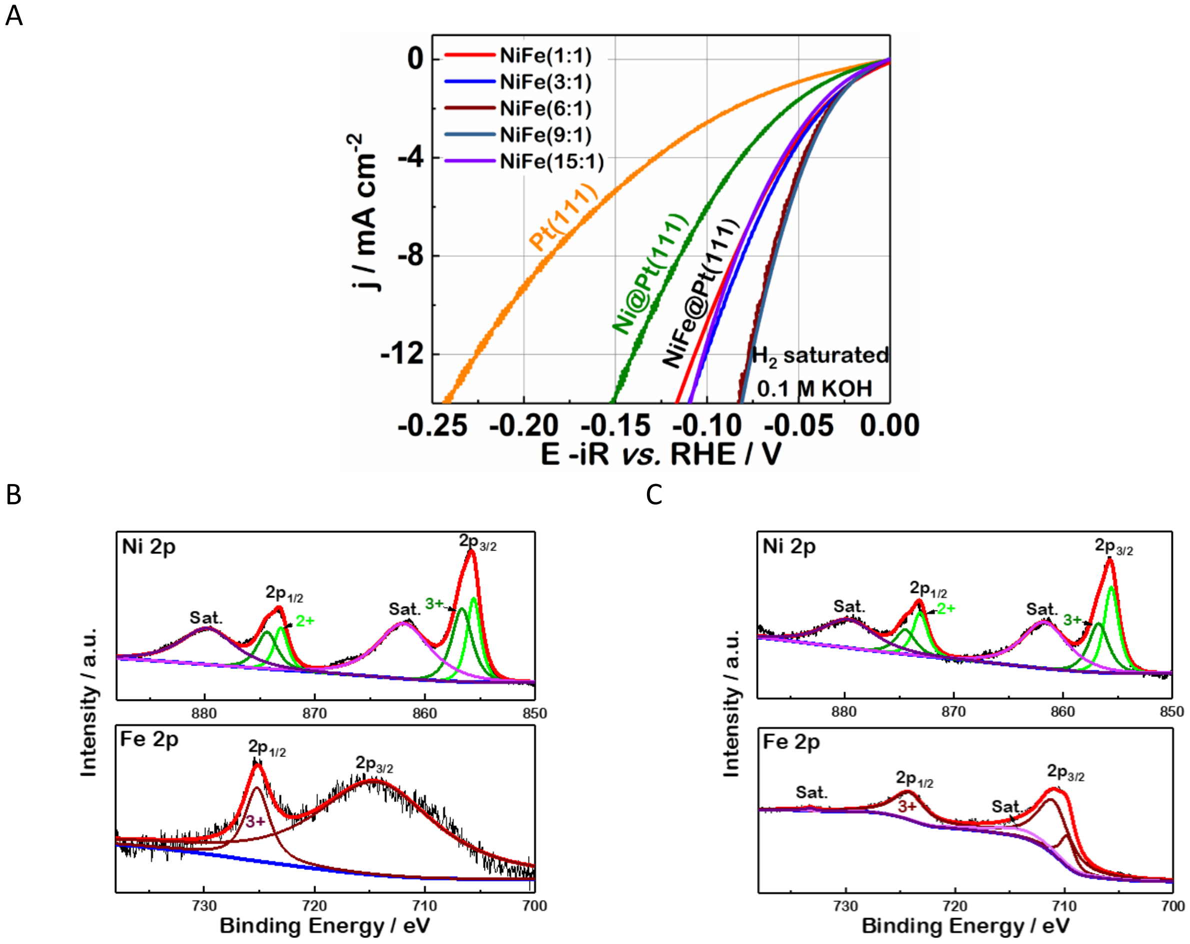

Figure S3. (A) RDE voltammograms of NiFe@Pt(111) electrodes, recorded in H_2-saturated KOH at a rotational speed of 1600 rpm, prepared by using precursor solutions with different ratios of Ni to Fe. The polarization curves were recorded at a scan rate of 50 mV s^{-1} and have been corrected by 85% of the IR-drop. Additionally, the activity of Pt(111) and Ni@Pt(111) is shown as reference. (B and C) Ni and Fe 2p XPS spectra of Pt(111) modified with 9:1 (B) and 3:1 (C) Ni:Fe ratio precursor solution.

Figure S4. STM images of NiFe@Pt(111) with different cluster coverages.

STM images of pure and modified P(111) were recorded to monitor and compare different Ni-Fe cluster coverages. The surface of pure Pt(111) is rather smooth (**Figure S4A**). At low precursor concentrations (100 times diluted), isolated or agglomerated Ni-Fe clusters with up to 3 nm in height appear on the Pt surface (**Figure S4B**). At "standard" precursor concentrations, cluster agglomerates with significantly increasing height can be observed (**Figure S4C**). Further increase of the concentration (1000 times higher) leads to the formation of a smooth Ni-Fe thin film (**Figure S4D**).

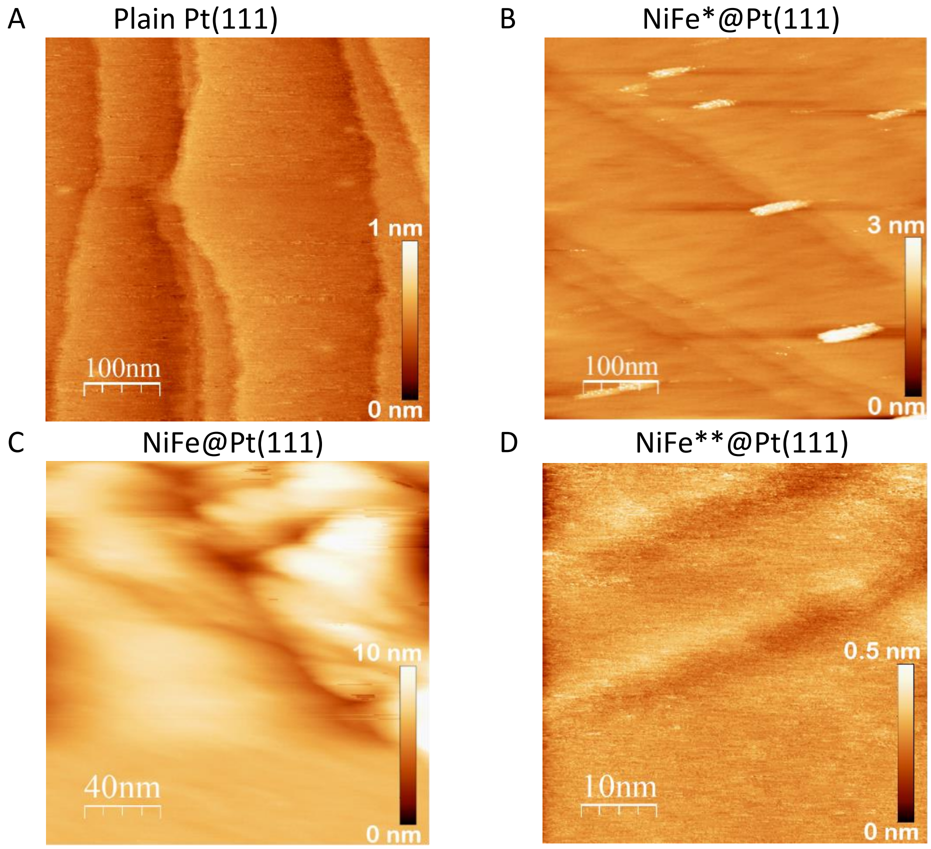

Figure S4. STM images of NiFe@Pt(111) with different cluster coverages: (A) plain Pt(111), (B-D) NiFe*@Pt(111), NiFe@Pt(111) and NiFe**@Pt(111), respectively.

Figure S5. SEM images of NiFe@Au-coated glass plates with different cluster coverages.

In order to further confirm the morphology of the Ni-Fe clusters, Au-coated glass plates modified with Ni-Fe clusters were studied by SEM. With the increase of the precursor concentration, agglomeration of the clusters takes place, consistent with observations from the STM studies.

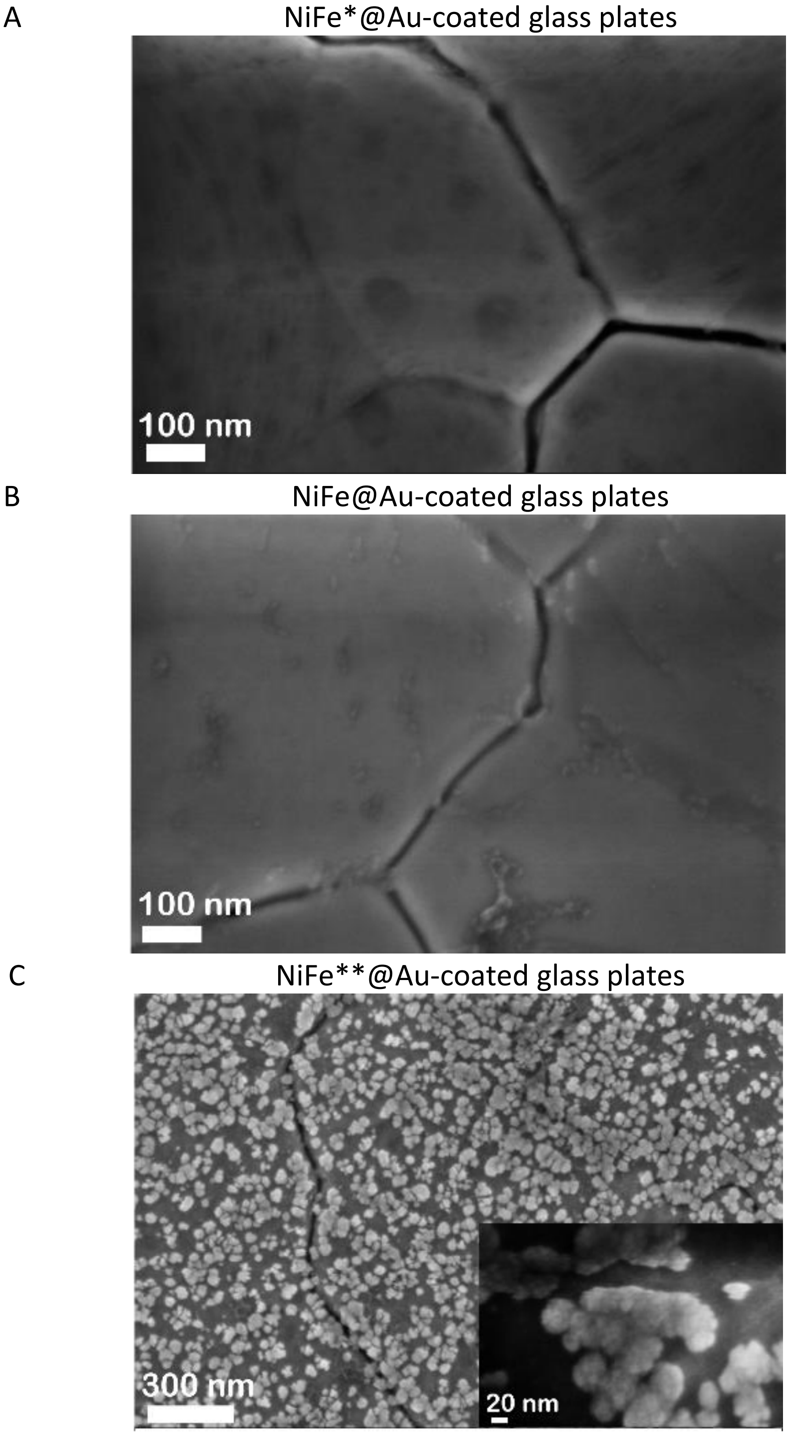

Figure S5. SEM images of (A) NiFe*@Au-coated glass plates, (B) NiFe@Au-coated glass plates and (C) NiFe**@Au-coated glass plates.

Figure S6. Tafel plots for HER on Pt(111) and NiFe@Pt(111) with different cluster coverages.

Tafel plots on Pt(111) and NiFe@Pt(111) prepared by different concentrations of precursor solutions were calculated from **Figure 2D**. At a relatively low overpotential, the Tafel slopes calculated from the polarization curves on both NiFe*@Pt(111) and NiFe@Pt(111) electrodes are 18 mV dec^{-1}, while on both NiFe**@Pt(111) and pure Pt(111) electrodes are 33 mV dec^{-1}. This drop in kinetic activity of the highest Ni-Fe coverage could be caused by a decreasing number of active sites on the Pt surface or by the comparatively low electrochemical activity of Ni/Fe atoms in multilayer non-noble metal hydroxides[7]. With increasing overpotential, the activity order observed is as follows: NiFe@Pt(111) > NiFe*@Pt(111) > NiFe**@Pt(111) > pure Pt(111). All the Tafel slopes calculated increase significantly due to mass transfer limitation, being 60 mV dec^{-1}, 80 mV dec^{-1}, 152 mV dec^{-1}, and 193 mV dec^{-1} for NiFe@Pt(111), NiFe*@Pt(111), NiFe**@Pt(111) and pure Pt(111), respectively. This difference in activity can be explained by the varying amounts of active Ni-Fe/Pt interfaces, which are too few in number at low surface coverages yet blocked at high surface coverages. These observations indicate that the HER performance of NiFe@Pt(111) is closely associated with the Ni-Fe cluster coverages on Pt(111).

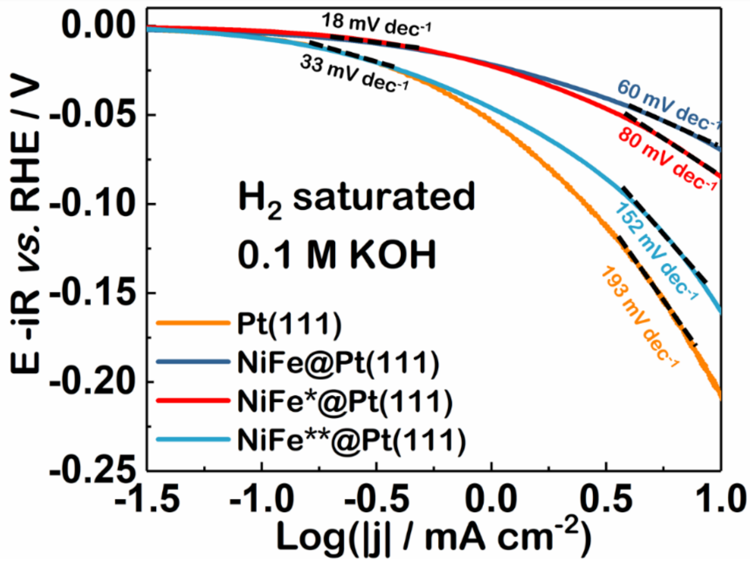

Figure S6. Tafel plots for HER on Pt(111) and NiFe@Pt(111) prepared by different concentrations of precursor solutions, under hydrogen atmosphere. The dash lines are fits of the curves from which the indicated Tafel slopes were obtained.

Figure S7. Comparison of OH_{ads} peaks for Pt(111) modified with Ni, Ni-Fe, Ni-Co clusters.

With regard to NiFe@Pt(111), both the onset-potential for OH_{ads} and the corresponding peak are negatively shifted with the increase of Fe percentage, relative to that of Ni@Pt(111). These results imply that the activities tuned by the Ni to Fe ratios are presumably through modifying the binding of *OH to NiFe@Pt(111), as this binding strength is closely associated with the amount of Fe in NiFe@Pt(111). Besides, compared with those of Ni@Pt(111) and NiFe@Pt(111), both the onset-potential for OH_{ads} and the OH_{ads} peak of NiCo@Pt(111) are obviously negatively shifted. These observations suggest that the binding of *OH to NiCo@Pt(111) is much stronger than those to Ni@Pt(111) and NiFe@Pt(111). It should be noted that the ratios shown here are the used precursor solution feeding ratios.

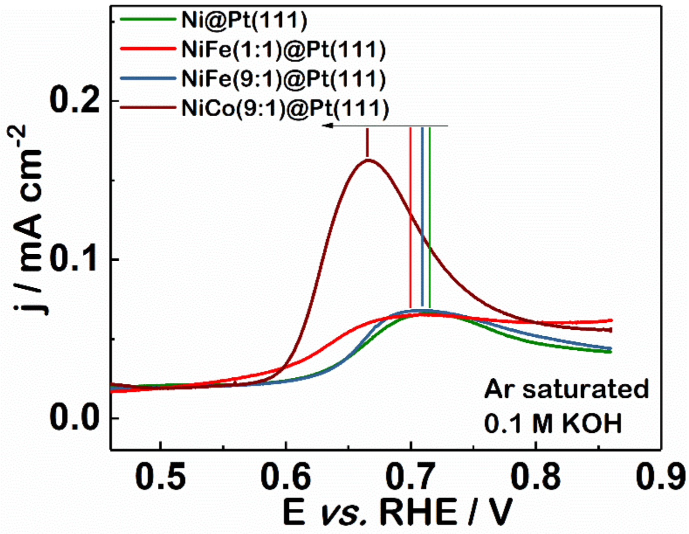

Figure S7. Comparison of OH_{ads} peaks for Pt(111) modified with Ni, Ni-Fe, Ni-Co clusters. Green: Ni@Pt(111), red: NiFe(1:1)@Pt(111), navy blue: NiFe(9:1)@Pt(111), dark red: NiCo(9:1)@Pt(111). Note that the ratios shown here are the used feeding ratios.

Figure S8. Comparison of OH_{ads} charge for Pt(111) modified with Ni, Ni-Fe, Ni-Co clusters.

In terms of NiFe@Pt(111), with the increase of Fe percentage, the OH_{ads} charge keeps increasing. This suggests that the binding of *OH to NiFe@Pt(111) can be tuned by changing the Fe amount in NiFe@Pt(111). Compared with those of Ni@Pt(111) and NiFe@Pt(111), the OH_{ads} charge of NiCo@Pt(111) is obviously increased. This phenomenon is associated with the relatively strong binding of *OH to NiCo@Pt(111).

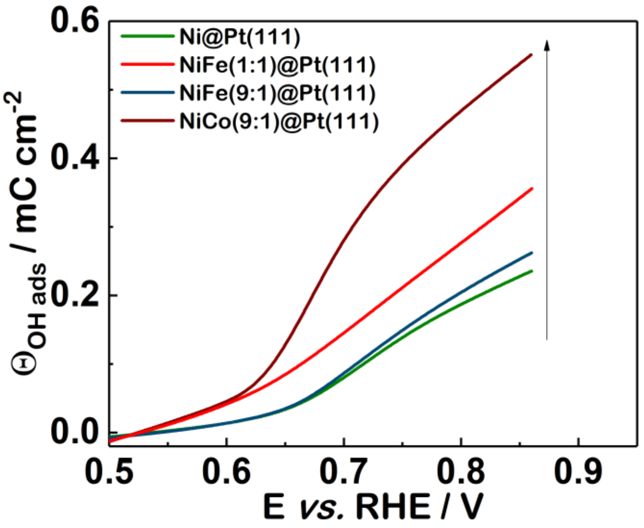

Figure S8. Comparison of OH_{ads} charge for Pt(111) modified with Ni, Ni-Fe, Ni-Co clusters. Green: Ni@Pt(111), red: NiFe(1:1)@Pt(111), navy blue: NiFe(9:1)@Pt(111), dark red: NiCo(9:1)@Pt(111). Note that the ratios shown here are the used feeding ratios.

Figure S9. “Volcano plot” of Pt modified with different metal hydroxide clusters.

A “volcano plot” of Pt modified by various metal hydroxide clusters is proposed to guide future catalyst design by comparing our work with data from the literature[2,8,9]. It indicates that the *OH binding energy can be a possible descriptor for alkaline HER activity. A weak *OH binding energy results in slow water dissociation, whereas a too strong *OH binding energy leads to *OH “poisoning”. Moreover, according to this guideline, adjusting the electronic band structure of Ni to find a more favorable balance appears to be a promising way of improving alkaline HER. However, it should be noted that more precise calculations of the *OH binding energy are still needed.

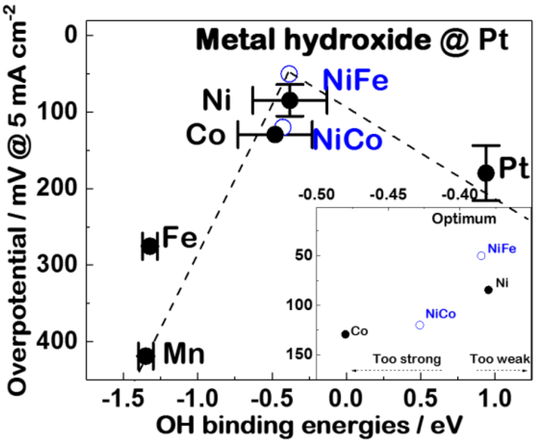

Figure S9. “Volcano plot” of Pt-modified with different metal hydroxide clusters: overpotential of HER in 0.1 M KOH at 5 mA cm^{-2} as a function of the *OH binding energy of the clusters deposited on Pt. The dashed line is a guide to the eye, indicating a possible trend towards optimum binding energy. The inset is a partial enlargement corresponding to the top of the volcano. Closed and open symbols refer to single and bimetallic hydroxide cluster modification, respectively. The *OH binding energies were calculated by density functional theory (DFT), taken from Ref.[8]. The corresponding activities were obtained from Ref.[2], [8] and [9], together with this work. For NiFe- and NiCo@Pt(111), it is difficult to distinguish the binding energy from that of Ni@Pt(111) using DFT calculations, due to the large degree of uncertainty. Consequently, their *OH binding energies were calculated from the potential shift of the OH_{ads} peaks relative to that of Ni@Pt(111) in the CVs. More details about this calculation can be found in Ref.[1] and [10].

Figure S10. Current-time plot obtained for the surface-modification process.

This chronoamperometry was the primary method used in this work for preparing the modified electrodes. The modification process was performed at a pre-set electrode potential of 0.1 V_{RHE} under a hanging meniscus configuration. The whole process was done under potential control and the used potential was much lower than the Pt oxidation potential, in order to safeguard the Pt surface.

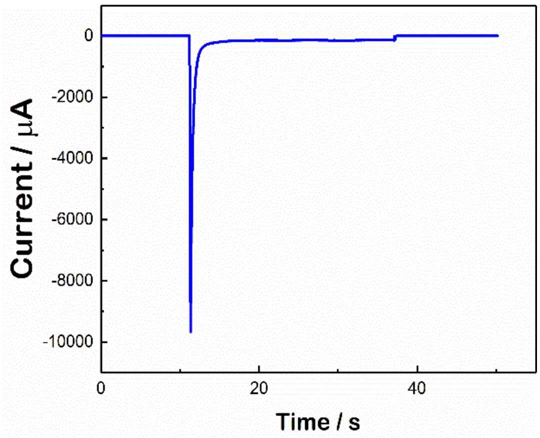

Figure S10. Current-time plot obtained for the surface-modification process. It was conducted under continuous application of 0.1 V_{RHE}, and details can be found in the experimental section.

Figure S11. Polarization curves of Ni@Pt/C and NiFe@Pt/C including both HER and HOR.

For the HOR, the obtained activity of NiFe@Pt is better than that of Ni@Pt. This observed activity trend is the same for the HER.

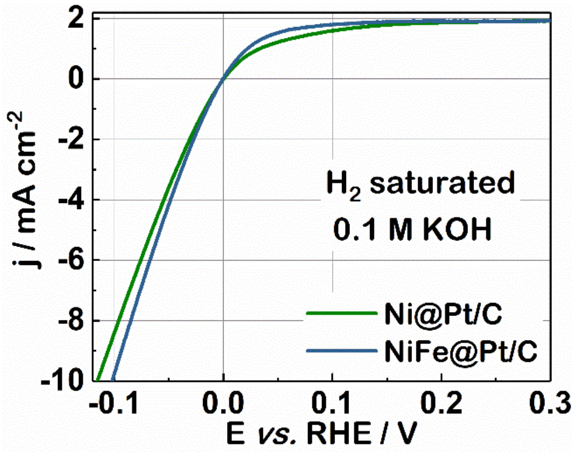

Figure S11. Polarization curves of Ni@Pt/C and NiFe@Pt/C measured in H_2 saturated 0.1 M KOH rotated at 1600 rpm.

Figure S12. Stability of NiFe@Pt(111).

During an accelerated durability test (we selected quite positive reverse potential of 0.4V to accelerate the corrosion) glassy carbon rod was used as the counter electrode to avoid a possible Pt deposition effect caused by the dissolution of the Pt counter electrode. The HER activity obtained at the 5th cycle is comparable to those using the Pt counter electrode, indicating an effect from Pt deposition can be neglected for shorter time periods. Moreover, the HER activity after 1000 cycles (~120 mV@10 mA cm-2) is still better than that of Ni@Pt(111) after only a few cycles (~128 mV@10 mA cm-2), even though it slightly decreases over time.

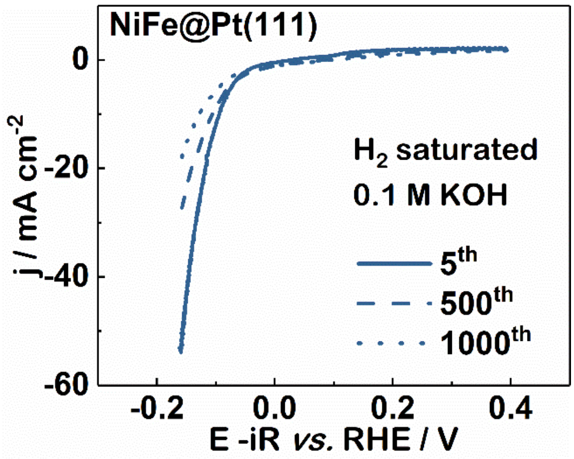

Figure S12. Stability of NiFe@Pt(111) measured by cyclic voltammetry. The HER polarization curves of the 5th, 500th and 1000th cycle were compared as shown by the solid, dashed and dotted line, respectively.

3. Supplementary References

[1] R. Subbaraman, D. Tripkovic, K. C. Chang, D. Strmcnik, A. P. Paulikas, P. Hirunsit, M. Chen, J. Greeley, V. Stamenkovic, N. M. Markovic, Nat. Mater. **2012**, *11*, 550.

[2] F. J. Sarabia, P. Sebastián-Pascual, M. T. Koper, V. Climent, J. M Feliu, *ACS Appl. Mater. Interfaces* **2018**, *11*, 613-623.

[3] X. Yu, J. Zhao, L. R. Zheng, Y. Tong, M. Zhang, G. Xu, C. Li, J. Ma, G. Shi, *ACS Energy Lett.* **2017**, *3*, 237-244.

[4] J. Li, S. Ghoshal, M. K. Bates, T. E. Miller, V. Davies, E. Stavitski, K. Attenkofer, S. Mukerjee, Z. Ma, Q. Jia, *Angew. Chem.* **2017**, *56*, 15594-15598.

[5] K. J. J. Mayrhofer, D. Strmcnik, B. B. Blizanac, V. Stamenkovic, M. Arenz, N. M. Markovic, *Electrochim. Acta* **2008**, *53*, 3181-3188.

[6] S. Zou, M. S. Burke, M. G. Kast, J. Fan, N. Danilovic, S. W. Boettcher, *Chem. Mater.* **2015**, *27*, 8011-8020.

[7] G. Wang, J. Parrondo, C. He, Y. Li, V. Ramani, *J. Electrochem. Soc*. **2017**, *164*, 1307-1315.

[8] R. Subbaraman, T. Dusan, D. Strmcnik, K. C. Chuang, U. Masanobu, A. P. Paulikas, V. Stamenkovic, N. M. Markovic, *Science* **2011**, *334*, 1256-1260.

[9] I. Ledezma-Yanez, W. D. Z. Wallace, P. Sebastián-Pascual, V. Climent, J. M. Feliu, M. T. M. Koper, *Nat. Energy* **2017**, *2*, 17031.

[10] I. E. Stephens, A. S. Bandarenka, F. J. Perez-Alonso, F. Calle-Vallejo, L. Bech, T. P. Johansson, A. K. Jespen, R. Feydenda, B. P. Knudsen, J. Rossmeisl, I. Chorkendorff, *J. Am. Chem. Soc.* **2011**, *133*, 5485-5491.
